# More and Less Fear in Serotonin Transporter Knockout Mice

**DOI:** 10.1111/gbb.70016

**Published:** 2025-02-07

**Authors:** João Lima, Marios C. Panayi, Trevor Sharp, Stephen B. McHugh, David M. Bannerman

**Affiliations:** ^1^ Department of Experimental Psychology University of Oxford Oxford UK; ^2^ Danish Research Centre for Magnetic Resonance (DRCMR), Department of Radiology and Nuclear Medicine Copenhagen University Hospital—Amager and Hvidovre Copenhagen Denmark; ^3^ School of Psychology University of New South Wales Sydney New South Wales Australia; ^4^ Department of Pharmacology University of Oxford Oxford UK; ^5^ Medical Research Council Brain Network Dynamics Unit Oxford UK

**Keywords:** anxiety, fear, inhibitory learning, Pavlovian conditioning, safety

## Abstract

Recent theories suggest that reduced serotonin transporter (5‐HTT) function, which increases serotonin (5‐HT) levels at the synapse, enhances neural plasticity and affects sensitivity to environmental cues. This may promote learning about emotionally relevant events. However, the boundaries that define such emotional learning remain to be established. This was investigated using 5‐HTT knockout (5‐HTTKO) mice which provide a model of long‐term elevated 5‐HT transmission and are associated with increased anxiety. Compared to wild‐type controls, 5‐HTTKO mice were faster to discriminate between an auditory cue that predicted footshock (CS+) and a cue predicting no footshock (CS−). Notably, this enhanced discrimination performance was driven not by faster learning that the CS+ predicted footshock, but rather by faster learning that the CS− cue signals the absence of footshock and thus provides temporary relief from fear/anxiety. Similarly, 5‐HTTKO mice were also faster to reduce their fear of the CS+ cue during subsequent extinction. These findings are consistent with facilitated inhibitory learning that predicts the absence of potential threats in 5‐HTTKO mice. However, 5‐HTTKO mice also exhibited increased generalisation of fear learning about ambiguous aversive cues in a novel context, different from the training context. Thus, 5‐HTTKO mice can exhibit both more and less fear compared to wild‐type controls. Taken together, our results support the idea that loss of 5‐HTT function, and corresponding increases in synaptic 5‐HT availability, may facilitate learning by priming of aversive memories. This both facilitates inhibitory learning for fear memories but also enhances generalisation of fear.

## Graphical abstract

We tested whether the loss of 5‐HTT function facilitates emotional learning due to increased sensitivity to emotionally relevant events and cues. Compared to wildtype littermates, 5‐HTTKO mice showed (i) increased sensitivity to aversive events (e.g., successive footshock deliveries), (ii) enhanced discriminative aversive learning, (iii) facilitated learning about the absence of predicted aversive events (e.g., safety cues during fear conditioning and extinction) and (iv) increased generalisation (i.e., across different contexts) of fear learning about ambiguous aversive cues.

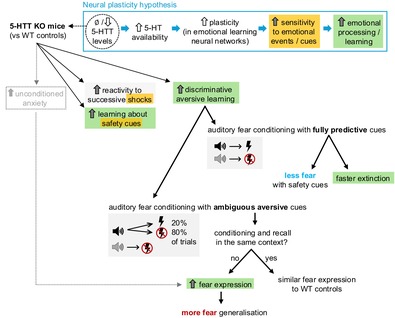

## 1 Introduction

Current theories suggest that serotonin (5‐HT) availability in the brain may not regulate mood directly but rather it may do so indirectly by promoting neural plasticity and facilitating emotional learning and sensitivity to emotionally relevant life events [1, 2, 3, 4, 5, 6, 7, 8, 9]. Therefore, the effects of increased 5‐HT on mood will depend on the background environment and the experiences of the individual [2, 3, 7]. However, within this neural plasticity hypothesis, the boundaries that define facilitated emotional learning remain to be fully established.

The availability of 5‐HT at the synapse is controlled by the serotonin transporter (5‐HTT), which is the target of selective serotonin reuptake inhibitors (SSRIs), the most commonly prescribed treatment for anxiety and depression. SSRIs act to increase synaptic levels of the neurotransmitter, which is thought to enhance neuronal plasticity and lead to changes in emotional processing and learning which can ultimately lead to an improvement in mood [4, 10, 11]. Paradoxically, studies in animals have long suggested that increased 5‐HT is associated with increased anxiety (e.g., [12, 13, 14, 15]). Furthermore, natural variation in the levels of the 5‐HTT has been reported in the human population [16, 17], and it has been suggested that this variation can influence emotional phenotypes, with lower 5‐HTT expression associated with an increased risk of developing anxiety and depression [6, 16, 18, 19, 20, 21]. However, it is important to point out that other studies do not support this narrative and meta‐analyses often fail to find links between the 5‐HTT and relevant behavioural and/or clinical phenotypes [20, 22, 23, 24, 25, 26].

Nevertheless, while findings in humans remain controversial, studies in animals have produced clearer results. Genetically modified rodents lacking the 5‐HTT provide an important model of long‐term elevated 5‐HT transmission [27, 28]. Notably, 5‐HTT knockout (5‐HTTKO) mice are more anxious than wild‐type controls and display increased behavioural despair in the forced swim test, whereas transgenic mice over‐expressing the transporter (5‐HTTOE mice) exhibit reduced anxiety [29, 30, 31, 32]. Importantly, 5‐HTTKO rodents have increased extracellular 5‐HT levels, whereas 5‐HTTOE mice have decreased levels [28, 33, 34].

Furthermore, variation in the levels of the 5‐HTT can also impact on fear learning [32, 35, 36, 37], a widely adopted model of emotional learning in rodents. Although early studies in 5‐HTTKO mice failed to see differences in fear acquisition during conditioning tasks when a single auditory cue which predicted footshock was used (e.g., [32]), we recently demonstrated enhanced fear discrimination learning in these knockout mice when they had to learn which of two distinct auditory cues predicted footshock and which cue predicted no footshock [38]. This was accompanied by increased neural activity in the amygdala in response to both of these emotionally relevant cues. Thus, lack of 5‐HTT activity, which is associated with increased 5‐HT availability, can lead to increased sensitivity to threat‐relevant cues and enhanced fear discrimination learning, at least under some circumstances (see also [39, 40, 41]). These data are consistent with the neural plasticity hypothesis, which suggests that 5‐HT promotes plasticity in key neural networks that underlie emotional learning ([42, 43, 44, 45]; however, see also [46, 47]).

A key question is whether 5‐HT facilitates all emotionally relevant learning and, if not, what are the boundaries for this enhanced learning? In the present study, we compared the performance of 5‐HTTKO mice and wild‐type controls on aversive Pavlovian fear discrimination tasks using both fully and partially predictive cues (conditioned anxiety), and unconditioned tests of anxiety. Our results suggest that increased 5‐HT availability can facilitate learning, at least in part by promoting priming of aversive memories, which can lead to either more or less fear depending on the experience of the animal.

## 2 Materials and Methods

### 2.1 Subjects

The 5‐HTTKO mice and wildtype controls, bred at the University of Oxford, were originally generated on a 129P1 (129P1/ReJ) × C57BL/6J hybrid background, before being repeatedly backcrossed onto a C57BL/6J background for more than eight generations [27]. The breeding strategy was the heterozygote mating of mice not closely related (i.e., not brothers or sisters). Multiple breeding pairs were used for generating experimental cohorts. As far as possible the KOs and WTs used in the experiments were littermates, originating from as many different breeding pairs as possible. Experiment 1 used 40 KOs (female: *n* = 20) and 42 wildtypes (female: *n* = 19) from 14 breeding pairs. Experiment 2 used 17 KOs (female: *n* = 7) and 18 wildtypes (female: *n* = 8) from 8 breeding pairs. Experiment 3 used 23 KOs (female: *n* = 12) and 23 wildtypes (female: *n* = 5) from 11 breeding pairs. A follow‐up study to Experiment 3 used 6 KOs (female: *n* = 3) and 5 wildtypes (female: *n* = 3) from 2 breeding pairs. The footshock reactivity test used 13 KOs (female: *n* = 7) and 18 wildtypes (female: *n* = 11) from 5 breeding pairs. Mice were at least 6 weeks old (most commonly 4–5 months; maximum 10 months) at the time of testing. Subjects were group‐housed with their littermates in enriched conditions in a temperature‐controlled colony room on a 12/12 h light/dark cycle (lights on at 7 am), with food and water always available. All mice were tested during the light phase.

### 2.2 Materials and Procedures

#### 2.2.1 Experiment 1: Unconditioned Anxiety

Two approach‐avoidance conflict tests were used to investigate unconditioned anxiety‐like behaviour: the elevated plus maze (EPM) and the hyponeophagia test (HPN; also known as novelty‐suppressed feeding). All subjects tested on the HPN test underwent the EPM test 1–2 days before. The EPM consisted of two sets of opposed arms (35 × 6 cm) perpendicularly united in a plus sign formation, elevated 70 cm above the floor. The ‘closed arms’ were enclosed by (20 cm high) walls, whereas the ‘open arms’ were just bordered by a slightly raised edge, which mice could grip. The maze was entirely grey‐coloured, with the open arm flooring having a lighter grey colour than the rest of the maze. Mice were placed at the end of a closed arm, facing away from the centre and allowed to explore the maze freely for 5 min. Luminance was set around 7 lx at the maze level and the flooring was cleaned with 10% alcohol between subjects. Behaviour was tracked with ANY‐maze Video Tracking Software (Stoelting Co., USA), which recorded an entry into an arm whenever 80% of the subject's body was inside that arm.

The apparatus used for the HPN test consisted of a translucent plastic jug (12.5 cm high, 18 cm long, 13 cm at its biggest width) placed upside down on a white plastic base (30 cm^2^; [48]). A protruding spout at one extremity of the jug (2 cm long) worked as an alcove over a food well (0.9 cm high, 1.2 cm in diameter) attached to the base. The experimental room had the luminance set around ~17 lx at the apparatus level. Sweetened condensed milk (Nestle Carnation diluted 50:50 with water) was poured into the food well immediately before testing. The mice had no prior experience of the sweetened condensed milk. After being food‐deprived in its home cage for 18–20 h and waiting 5–20 min in an individual cage inside the experimental room, a mouse was placed on the base facing away from the food well while the jug was gently lowered. The mouse was left to move freely either until the start of continuous drinking (~3 s) or until 3 min elapsed. If continuous drinking did not occur during this period, the subject was returned to its individual cage for 10–12 min, before being tested again in the same conditions, in a test–rest–test cycle [48]. A maximum of three trials was given to each subject.

#### 2.2.2 Experiments 2 and 3: Fear Conditioning

Mice were tested in two enclosed conditioning chambers (ENV‐307A, Med Associates Inc., USA), each associated with distinct visual and olfactory cues (wallpaper patterns on sound‐attenuating cubicles: plain white or black and white stripes; scent: almond or lavender). Stimuli were administered via Med‐PC IV software (Med Associates; https://med‐associates.com/product/med‐pc/; RRID:SCR_012156) and sessions were video‐recorded using a miniature camera with a wide‐angle lens placed above the chambers. Subjects were fear conditioned for 2 days (Conditioning days 1 and 2, or C1 and C2) in one context, and then, over the next 3 days, subjected to a Fear Memory Recall session (FMR) and then further extinction training (Extinction days 1 and 2, or E1 and E2) conducted in the alternative context (unless otherwise stated). Technically, these last three sessions were all extinction sessions but, in practice, the first one (FMR) also works as a recall test given the reduced number of non‐reinforced CS+ trials administered. Contexts were counterbalanced across mice.

Experiment 2 used a discriminative fear conditioning task, in which mice were required to discriminate one auditory cue (e.g., CS+ = 2900 Hz tone) that was always followed by a mild footshock (0.3 mA, 0.5 s) from another auditory cue that was never paired with footshock (e.g., CS− = white noise; see Figure S1A,C). Both cues were 30 s in duration and delivered at 72 dB. Allocation of the 30 s‐long tone and white noise cues as the CS+ and CS− was counterbalanced across mice. Thus, for approximately half of the wildtypes and half of the KOs the tone served as the CS+ cue and the white noise acted as the CS− cue. For the remaining mice this allocation was reversed (i.e., white noise is CS+ and tone is CS−). In any given session, after a lead‐in period to the first cue presentation (5 min), mice were exposed to 5 presentations of each cue type in a pseudo‐randomly, interleaved order. A mean inter‐cue interval of 80 s (range 60–100 s) was used. No footshocks were given during the FMR and extinction sessions.

Mice used in Experiment 3 were exposed to the same discriminative task design and parameters as those used in Experiment 2, with the exception that the CS+ cue was replaced with a cue that only predicted footshock on 20% of the trials (i.e., an ambiguous CS, or CSa; see Figure S1B,C). Footshocks were delivered with the third CSa presentation of Conditioning day 1, and the second CSa presentation of Conditioning day 2.

Following Experiment 3, a further cohort of experimentally naïve mice was tested in the same protocol as Experiment 3 but with one alteration: the FMR session was now conducted in the same conditioning chamber as used for fear acquisition (see Figure S1B,D). Combined with Experiment 3, this task was used to assess whether genotypes differed regarding the generalisation of cue discrimination learning with ambiguous aversive cues across different contexts.

#### 2.2.3 Footshock Reactivity Test

Finally, to determine whether any genotypic differences observed in Experiments 2 and 3 were due to altered sensitivity to footshock in the 5‐HTTKO mice, a footshock reactivity test was administered to another cohort of naïve mice. Five different footshock intensities were used (0.05, 0.1, 0.2, 0.3 and 0.4 mA), each one presented two times in the same test session (see Figure S1E). Specifically, after a 5 min lead‐in period, the first five footshocks were administered in ascending order of intensity, and the last five in descending order (mean inter‐shock interval: 50 s; range: 30–70 s). The test was administered in the same apparatus used for Experiments 2 and 3, with footshocks also lasting 0.5 s.

### 2.3 Data Analyses

#### 2.3.1 Unconditioned Anxiety

Latency to first entry to an open arm, % of time spent in open arms ([‘time in open arms’/‘total time spent in all arms’] × 100), and total distance travelled were investigated in the EPM. In the HPN test, latency to (i) first contact (including sniffs) with the milk, and (ii) begin continuous drinking were recorded by the experimenter from watching recorded videos, blinded to the genotype. For example, if the mouse displayed continuous drinking after 62 s then it was given a latency score of 62 s and was not re‐tested. However, if the mouse failed to drink within 180 s, then it was removed from the apparatus and returned to the home cage for 10–12 min. The mouse was then re‐tested under the same conditions in a second three‐minute trial. If the mouse exhibited continuous drinking after 30 s of trial 2 it was assigned a latency to continuous drinking of 210 s (i.e., 180 s of trial 1 plus 30 s of trial 2). If the mouse failed to drink during trial 2 it was again removed from the apparatus and returned to its home cage for a further 10–12 min before a third trial under the same conditions. If the mouse began continuous drinking after 30 s of trial 3 it was assigned a latency to continuous drinking of 390 s (i.e., 180 s of trial 1 plus 180 s of trial 2 plus 30 s of trial 3).

#### 2.3.2 Fear Conditioning

The measure used to assess acquired fear in the fear conditioning experiments was conditioned freezing behaviour. Freezing was assessed by processing each video recording on an automated movement detection software using a script in NIH Image (https://imagej.net/nih‐image/about.html; RRID:SCR_003073; [49]). This software compared consecutive video frames (1 Hz sampling) for pixel changes and assigned a freezing score (per each second of analysis) if the % pixel change was below a set threshold calibrated for an absence of movement except for breathing [50]. Previous studies revealed a 90% correspondence between this automated scoring method and human visual scoring for freezing. Conditioned freezing was calculated by selecting the freezing scores of each CS period and dividing the number of seconds where freezing occurred (e.g., 23) by the total duration of that period (i.e., 30), multiplied by 100 (e.g., (23/30) × 100 = 76.7%). In addition, a similar quantification was performed for the 30 s‐long (baseline) period prior to each CS onset (pre‐CS period). CS evoked changes in freezing (Δfreezing) were then calculated for each CS presentation by subtracting the % of freezing obtained during the pre‐CS period from the levels observed during the CS period. This is a bidirectional measure as CS‐evoked freezing greater or lower than the pre‐CS period levels yields positive or negative scores, respectively. Additionally, a discrimination (learning) index incorporating pre‐CS periods was also calculated daily for each group by subtracting the mean CS− Δfreezing score from the mean CS+ Δfreezing score. Analyses typically involved averaging the behavioural responses over all trials of an experimental session first, but comparisons between cues within the one session only were also made on Conditioning Day 1 (C1) of Experiment 2 (i.e., the first CS+ of the session was compared to the first CS− presented, and so on). This was possible as the pseudo‐random order of cue presentations made every two presented cues a CS+ and CS−.

#### 2.3.3 Unconditioned Responses and Reactivity to Footshock

Responses to footshock deliveries in Experiments 2 and 3 and in the footshock reactivity test relied on using the % pixel changes in each 1 s‐long bin, computed by the NIH Image software, as an index of activity levels. Specifically, responses were obtained by calculating the change in activity levels evoked by a footshock delivery (i.e., Δactivity) during a post‐shock 5 s period compared to the pre‐shock, local baseline period (Δactivity (%) = mean % pixels changed during the 5 s‐long post‐shock period minus mean % pixels changed during a 5 s‐long period before the footshock). The larger the % pixel change, the higher the burst in activity elicited by the footshock. The local baseline in the footshock reactivity task was taken immediately before the 1 s‐long bin containing the footshock delivery, whereas in the fear conditioning tasks it was the 5 s period preceding the CS that co‐terminated with the footshock delivery. The latter selection more closely resembles the baseline periods used in the footshock reactivity test, which were not affected by auditory cues. In the footshock reactivity test, the data are presented as the mean response to the two footshock presentations of each intensity.

### 2.4 Statistical Analysis

Data obtained with approach‐avoidance conflict tests were analysed with independent samples t‐tests or, when parametric approaches could not be used, a Mann–Whitney *U* test. Data obtained with Experiments 2 and 3 and follow‐up studies (investigating genotypic differences in fear generalisation across different contexts and footshock reactivity) were analysed using analysis‐of‐variance (ANOVA) in SPSS (version 22; IBM, Armonk, New York, USA; https://www.ibm.com/products/spss‐statistics; RRID:SCR_016479). ANOVAs are described in the form: A_2_ × B_3_, where A is a factor with two levels and B a factor with three levels. A factor of Experiment was included in the analyses of the unconditioned response to the first footshock (US #1), which included the combined data from Experiments 2 and 3.

ANOVAs with an additional factor of sex for all experiments did not significantly alter the effects reported here, and there were no significant Genotype × Sex interactions. To help the readability of the manuscript, the factor of sex is therefore not reported. The sphericity assumption was verified by means of the Mauchly's test and, when violated, the degrees of freedom for the *F* values were adjusted via the Greenhouse and Geisser correction. When the correction factor ε was near or above 0.75, then a Huynh and Feldt correction was used instead [51]. However, in the interests of clarity, the original degrees of freedom have been reported throughout the main text when a correction was made.

In Experiment 3, data from Conditioning Day 1 are shown in the figures but were not included in the statistical analyses as mice had little opportunity to learn about the meaning of the CSa during this session (i.e., they were subjected to only one footshock delivery for 10 cue presentations).

Bar charts depicting the mean and standard error of the mean (SEM) were used to graph the discrimination index results from Experiments 2 and 3, as well as responses to footshock. Information about individual data points is provided in Figure S2.

## 3 Results

### 3.1 Experiment 1: Increased Unconditioned Anxiety in 5‐HTTKO Mice

In agreement with previous studies [29, 31], 5‐HTTKO mice exhibited increased levels of unconditioned anxiety compared to wildtype controls (see Table 1). They were slower to enter and spent less time overall in the open arms of the elevated plus maze. They were also less active overall during the EPM test (total distance travelled; see also [31]). 5‐HTTKO mice were also slower to start drinking the highly palatable, sweetened condensed milk in the hyponeophagia test although, importantly, this did not reflect a difference in latency to first contact with the milk. Thus, 5‐HTTKO mice exhibited behaviours consistent with increased unconditioned anxiety.

**TABLE 1:** Elevated plus maze (EPM) and hyponeophagia (HPN) tests for wildtype (WT) and 5‐HTTKO mice.

Test	Measure	WT	KO	Statistics
Elevated plus maze	Latency to first OA entry	61.9 + 11.9 (30.6 [19.7–59.5])	*145.9 + 20.6 (73.8 [25.9–300])	*U* = 566, *p* = 0.011
	% of time in OA	7.6 + 0.9 (7.2 [2.9–11.1])	**3.5 + 0.7 (1.8 [0–5.7])	*U* = 445.5, *p* < 0.001
	Total distance travelled	12.1 + 0.4	**8 + 0.4	*t*(80) = 7.3, *p* < 0.001
Hyponeophagia	Latency to first contact with the milk	4.7 + 1.2 (2.4 [1–6])	3.6 + 1.1 (2 [1–4])	*U* = 279.5, *p* = 0.521
	Latency to continuous drinking	34.4 + 13.6 (10.9 [7.5–28])	*61.6 + 24 (25.5 [13.7–41])	*U* = 209.5, *p* = 0.046

*Note:* Mice tested on the EPM: WTs = 42; KOs = 40. Mice tested on the HPN test: WTs = 25; KOs = 25. 5‐HTTKO mice exhibited increased anxiety‐like behaviour during both tests. Latencies are indicated in seconds. Total distance travelled is indicated in metres. Mean ± SEM, *p < 0.05, **p < 0.001. Medians and interquartile ranges are in parentheses for results analysed by a Mann–Whitney *U* test.

### 3.2 Experiment 2: 5‐HTTKO Mice Exhibit Enhanced Discriminative Aversive Learning

**FIGURE 1:**
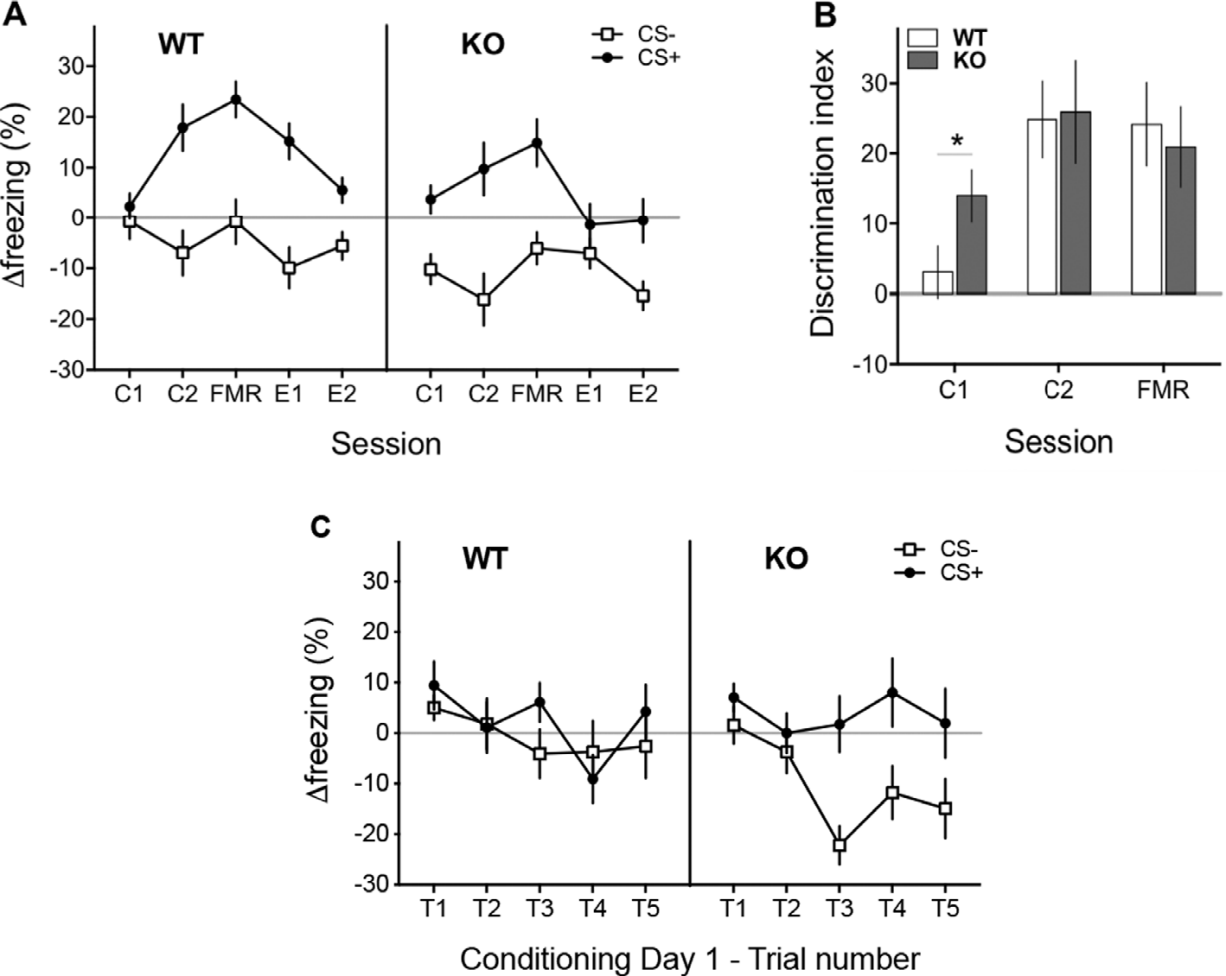
Behavioural responses during the discriminative aversive learning task used in Experiment 2 with wildtype (WT) and 5‐HTTKO (KO) mice (N sizes: WT = 18; KO = 17). (A) Cue‐evoked changes in freezing relative to pre‐cue freezing levels (Δfreezing) on the Conditioning (C1, C2), Fear Memory Recall (FMR) and further Extinction (E1, E2) sessions. FMR, E1 and E2 were administered in a different context from the one used in C1 and C2. Both genotypes learned to discriminate between the two cues, with the CS+ evoking an increase in freezing compared to the pre‐CS period, and the CS− a decrease in freezing compared to baseline. (B) Discrimination index (CS+ Δfreezing—CS− Δfreezing) during C1, C2 and FMR. Discriminative aversive learning was faster in the 5‐HTTKO mice, as evidenced by the significantly higher discrimination index seen in these mice compared to WT mice on C1. (C) Cue‐evoked Δfreezing, compared to pre‐cue periods, for each successive pair of CS+ and CS− presentations (i.e., for each trial; T1–5) during C1. The enhanced fear discrimination learning seen in 5‐HTTKO mice developed during C1 and was driven by a decrease in freezing during the CS− presentations compared to the pre‐CS periods. Mean ± SEM, *p < 0.05.

In agreement with our recent study [38], both genotypes rapidly acquired the CS+/CS− discrimination but 5‐HTTKO mice exhibited faster discrimination learning than wildtype mice. Analysis of Δfreezing scores (ANOVA model: Genotype_2_ × Day_5_ × CS_2_) revealed that the CS+ evoked more Δfreezing than the CS− (main effect of CS type: *F*(1,33) = 43.2, *p* < 0.001). However, the pattern of responding to the cues in the two genotypes differed significantly over the 5‐day procedure (Genotype_2_ × Day_5_ × CS_2_ interaction: *F*(4,132) = 3.9, *p* = 0.005; see Figure 1A). Importantly, 5‐HTTKO mice showed significantly stronger fear discrimination on Conditioning Day 1 (C1) compared to wildtype mice (discrimination index; simple effect of genotype on C1: *F*(1,33) = 4.3, *p* = 0.045; see Figure 1B). Subsequently, both groups showed similar discrimination levels during the C2 and FMR sessions (C2 and FMR; *F'*s < 0.149, *p'*s > 0.702). Analysis of freezing levels during the pre‐CS periods (ANOVA model: Genotype_2_ × Day_3_ × pre‐CS_2_) did not differ significantly between genotypes during the first 3 days (*F'*s < 1.3, *p'*s > 0.268).

### 3.3 Enhanced Fear Discrimination in 5‐HTTKO Mice Is Driven by CS– Responses

Trial‐by‐trial cue‐evoked Δfreezing scores from Conditioning Day 1 showed that the enhanced cue discrimination seen in 5‐HTTKO mice developed throughout this first session (Figure 1C). Both genotypes exhibited no cue discrimination during trials 1 and 2 (T1‐T2). However, from the third trial onwards, 5‐HTTKO mice showed better discrimination between the cues with a stronger reduction of freezing during the CS− presentations than wildtype mice (i.e., more movement than in the pre‐CS period). Notably, statistical analyses across the five trials of Conditioning Day 1 (ANOVA model: Genotype_2_ × Trial_5_ × CS_2_) confirmed that the main genotypic difference was predominantly in the CS− Δfreezing (Genotype_2_ × CS_2_ interaction: *F*(1,33) = 4.3, *p* = 0.045; simple main effect of genotype on CS− Δfreezing: *F*(1,33) = 4.2, *p* = 0.050; CS+ Δfreezing: *F*(1,33) = 0.14, *p* = 0.713). Differences in pre‐CS freezing levels during Conditioning Day 1 cannot explain this genotypic difference (*F'*s < 1.02, *p'*s > 0.409). Thus, the enhanced discrimination learning seen in 5‐HTTKO mice early in training was driven by differences in CS− Δfreezing and not by responses to the CS+ cue.

### 3.4 5‐HTTKO Mice Exhibit Enhanced Extinction of Cue Discrimination

**FIGURE 2:**
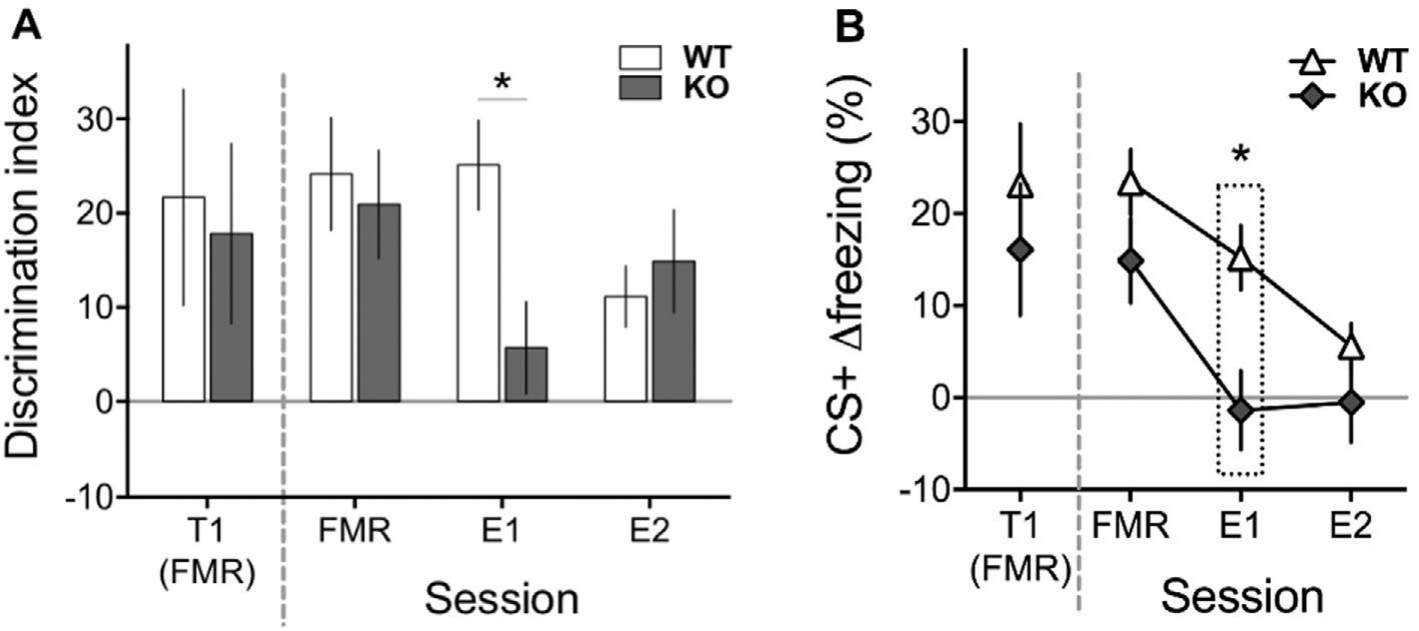
Behavioural responses during the Fear Memory Recall (FMR) and Extinction sessions (E1, E2) of Experiment 2, in a different context from conditioning, in wildtype (WT) and 5‐HTTKO (KO) mice (N sizes: WT = 18; KO = 17). The FMR session is also performed in extinction. (A) Discrimination index (CS+ Δfreezing—CS− Δfreezing) during the FMR, E1 and E2 sessions. T1 is the first CS+/CS− discrimination of the FMR session. Extinction learning was facilitated in the 5‐HTTKO mice, as evidenced by the significantly reduced discrimination index seen in these mice, compared to WT mice, during E1. (B) CS+ Δfreezing during T1, FMR, E1 and E2. The CS+ Δfreezing was significantly lower in 5‐HTTKO mice on E1, indicating that this decrease in responding to the CS+ underpins the significantly reduced cue discrimination seen in these mice on that session. The mean discrimination index on FMR, and the mean CS+ Δfreezing on FMR, E1 and E2 are re‐presented here from Figure 1. Mean ± SEM, *p < 0.05.

By the end of fear conditioning training both groups displayed strong and equivalent discrimination between the two auditory cues. Furthermore, equivalent discrimination levels were seen during the FMR session conducted in a different context (effectively the first day of extinction), including on the very first trial of that session (*t*(33) = 0.25, *p* = 0.801; see Figure 2A). However, the genotypes then differed across two further days of extinction training with faster extinction in the 5‐HTTKO mice. Specifically, analysis of the discrimination index data across these three sessions (FMR, E1 and E2; ANOVA model: Genotype_2_ × Day_3_) revealed that the 5‐HTTKO mice now exhibited significantly poorer discrimination than wildtype controls on E1 (Genotype_2_ × Day_3_ interaction: *F*(2,66) = 5.0, *p* = 0.010; simple effect of genotype on E1: *F*(1,33) = 8.1, *p* = 0.007; see Figure 2A). Further analysis demonstrated that these genotypic differences in extinction learning reflected how the two genotypes differed in their Δfreezing responses specifically to the CS+ cue (Figure 2B). 5‐HTTKO mice exhibited significantly lower CS+ Δfreezing during E1 compared to wildtype mice (effect of genotype on E1: *F*(1,33) = 9.1, *p* = 0.005). These results suggest facilitated extinction learning in 5‐HTTKO mice.

### 3.5 Experiment 3: 5‐HTTKO Mice Are More Likely to Generalise Fear Memory to a Novel Context

In the real world, many cues that become associated with threat stimuli are ambiguous and, therefore, unreliable predictors of aversive events, thus generating conditioned anxiety [52, 53, 54, 55, 56]. We have shown previously that discrimination between an ambiguous cue that sometimes predicts footshock and a cue that predicts no footshock is particularly affected in 5‐HTTOE mice [57]. We next assessed the behaviour of the 5‐HTTKO mice on an ambiguous cue fear conditioning task.

**FIGURE 3:**
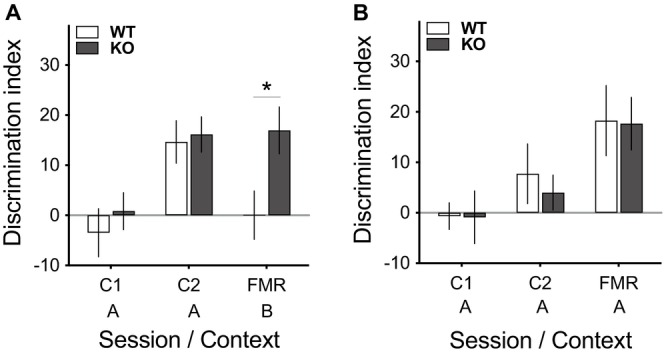
Behavioural responses during the discriminative aversive learning task used in Experiment 3 with an ambiguous CS cue (CSa) and a CS− cue in wildtype (WT) and 5‐HTTKO (KO) mice (A: Recall in a different context from conditioning; B: Recall in the same context as conditioning). X‐axis: Sessions were Conditioning day 1 (C1), Conditioning day 2 (C2), Fear Memory Recall (FMR), conducted in either Context A or B. (A) Discrimination index (CSa Δfreezing—CS− Δfreezing) during C1, C2 and FMR (novel context). Although both groups learned the CSa/CS− discrimination by C2, only 5‐HTTKO mice showed cue discrimination when tested for FMR in a novel context. (N sizes: WT = 23; KO = 23) (B) Discrimination index during C1, C2 and FMR. The data indicate that both WT and KO mice show strong cue discrimination levels when tested for FMR in the conditioning context. (N sizes: WT = 5; KO = 6). Mean ± SEM, *p < 0.05.

While both genotypes acquired the CSa/CS− discrimination at a similar rate, only 5‐HTTKO mice expressed this learning during a FMR session conducted in a different context (Figure 3A). Data from the first conditioning day (C1), which reflect just a single tone‐shock pairing, were not included in the ANOVAs. On the following day (C2), both genotypes exhibited equivalent and significant cue discrimination in freezing levels. But when fear memory was then subsequently tested in a novel context (i.e., different from the training context), only the 5‐HTTKO mice expressed this learned discrimination as increased freezing to the CSa compared to the CS− (discrimination index: ANOVA model: Genotype_2_ × Day_2_; Genotype × Day interaction: *F*(1,44) = 4.5, *p* = 0.039; see Figure 3A). Accordingly, 5‐HTTKO mice showed significantly greater discrimination than wildtype mice during FMR (simple effect of genotype on FMR: *F*(1,44) = 6.3, *p* = 0.016), even though the genotypes had not differed in their learning in the conditioning context (C2: *F*(1,44) = 0.074, *p* = 0.786). Furthermore, in the FMR session, the CSa and CS− evoked equivalent levels of Δfreezing in wildtype mice, resulting in no overall discrimination, but there was strong cue discrimination in the KOs (simple effect of CS for each genotype: wildtypes: *F* < 1; KOs: *F*(1,44) = 12.7, *p* = 0.001). Notably, this differs from the behaviour of the mice on C2, during which both groups were capable of significant discrimination between the cues (simple effect of CS for each genotype: wildtypes: *F*(1,44) = 14.1, *p* = 0.001; KOs: *F*(1,44) = 17.1, *p* < 0.001). Differences in pre‐CS freezing levels cannot explain the genotypic differences (*F'*s < 2.1, *p'*s > 0.156).

Although this enhanced discrimination performance seen in 5‐HTTKO mice during recall could reflect better fear learning, this was not evident on the conditioning days. An alternative possibility is that these mice may be more likely to generalise fear memory associated with a learned threat cue to a novel context. This seems a more likely possibility given the identical performance levels during C2 in both genotypes. This would predict normal levels of fear expression if mice were tested in the same context as used in conditioning. We, therefore, investigated the effect of context on fear memory recall using this same ambiguous cue conditioning paradigm but now testing recall in the ‘same’ context as used in conditioning. Importantly, both genotypes now showed robust and equivalent discrimination performance when recall was tested in the conditioning context (ANOVA model: Genotype_2_ × Day_2_ × CS_2_; main effect of CS type: *F*(1,9) = 12.0, *p* = 0.007; main effect of day: *F*(1,9) = 5.1, *p* = 0.050; interactions between genotype, day and CS type: *F*s < 1; still, simple effect of CS type on FMR: wildtypes: *p* = 0.018; KOs: *p* = 0.013; see Figure 3B). Thus, the data indicate that both wildtype and 5‐HTTKO mice can show CSa/CS− discrimination if the FMR session takes place in the conditioning context. In contrast, only 5‐HTTKO mice expressed this learning when recall was assessed in a novel context. Thus, 5‐HTTKO mice were more likely than wild‐types to generalise learning about threat cues across different contexts when an ambiguous aversive cue was used.

### 3.6 5‐HTTKO Mice Show a Normal Unconditioned Response to the First Footshock but Increased Reactivity to Successive Footshock Deliveries

**FIGURE 4:**
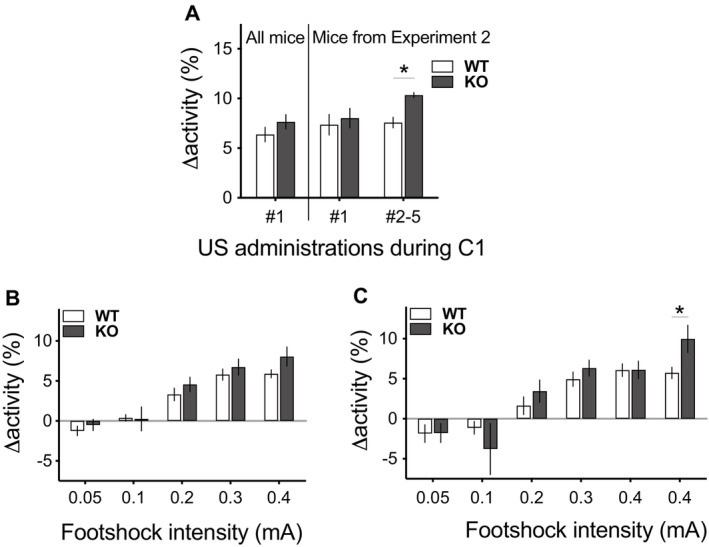
Responses to the unconditioned stimulus (US, i.e., a 0.5 s‐long, 0.3 mA footshock) in mice fear conditioned in Experiments 2 and 3 (A) and responses to five different shock intensities (0.05, 0.1, 0.2, 0.3 and 0.4 mA) in mice subjected to a footshock reactivity test (B, C). The responses were measured as mean changes in activity level (Δactivity (%) = mean activity level during the 5 s‐long post‐shock period minus mean activity level during a same‐length pre‐shock baseline period). (A) The first two bars on the left show the Δactivity for the first footshock delivery (i.e., US #1) of Conditioning Day 1 (C1) in the wildtype (WT) and 5‐HTTKO (KO) mice used in Experiments 2 and 3 (‘All mice’: WT = 41; KO = 40). The following bars show the changes in Δactivity for footshocks paired with the CS+ during C1 (US #1–5) in the mice tested only in Experiment 2 (WT = 18; KO = 17). No significant genotypic differences were found regarding the unconditioned response to the first footshock (i.e., to US #1), but 5‐HTTKO mice exhibited greater footshock reactivity across the remaining four footshocks of the fear conditioning session (i.e., USs #2–5). (B) Mean changes in activity level evoked by the two presentations of each footshock intensity used in a footshock reactivity test (N sizes: WT = 18; KO = 13). Genotypes did not significantly differ on their mean reactivity to any of five footshock intensities tested. (C) Mean changes in activity level evoked by each of the first six footshocks administered during the footshock reactivity test, in the order of presentation. Although no genotypic differences were seen in the first presentation of each distinct footshock intensity, the successive presentation of two 0.4 mA footshocks led to a significantly stronger reactivity to the second shock in 5‐HTTKO mice, compared to WT mice. Mean ± SEM, *p < 0.05.

Finally, we examined the behavioural responses of the mice to the aversive shock stimuli. This response was evident as a brief burst of locomotor activity. Analysis of the unconditioned response to the very first footshock delivery on C1 (i.e., US #1) for mice that underwent fear conditioning in Experiments 2 and 3 (ANOVA model: Experiment_2_ × Genotype_2_) revealed no statistically significant genotypic differences in the subjects' shock‐elicited activity levels (main effect of genotype: *F*(1,77) = 1.4, *p* = 0.248; see Figure 4A). Moreover, genotypes also did not differ in their pre‐US activity levels (ANOVA model: Experiment_2_ × Genotype_2_; main effect of genotype: *F* < 1).

However, the response to a further footshock in an already highly fearful or anxious animal that has already received a footshock in that current situation might be very different from the UR to a novel and completely unexpected footshock. A further analysis focusing on the data from Experiment 2, in which multiple footshocks were delivered in a session, showed that subsequent presentation of further footshocks throughout the C1 session (USs #2–5) led to differences in how the two genotypes reacted to the footshocks (see Figure 4A). 5‐HTTKO mice now exhibited greater reactivity levels than wildtype mice across the remaining four footshocks in the session (ANOVA model: Genotype_2_ × Trial_4_; main effect of genotype: *F*(1,33) = 6.1, *p* = 0.019). Genotypes did not differ in their pre‐US activity levels during presentation of further footshocks on C1 (ANOVA model: Genotype_2_ × Trial_4_; main effect of genotype and interaction between genotype and trial: *F* < 1).

We next investigated whether there was altered sensitivity to the footshock in 5‐HTTKOs compared to wildtype mice, across a range of footshock intensities in separate, experimentally naïve cohorts of wild type and 5‐HTTKO mice. Data analyses of the footshock reactivity test revealed that the genotypes did not differ in their mean reactivity to five different footshock intensities (averaged across two presentations of each intensity in an ascending/descending design). Analysis of the mean changes in activity levels evoked by each footshock (i.e., Δactivity, where an increased burst of activity reflects an increased footshock reactivity; ANOVA model: Genotype_2_ × Intensity_5_), confirmed that higher intensities of footshock evoked greater Δactivity in all mice (main effect of intensity: *F*(4,116) = 42.6, *p* < 0.001; see Figure 4B). However, the two genotypes were fairly well matched in their reactivity to all footshock intensities used (Genotype_2_ × Intensity_5_ interaction and main effect of genotype: *F'*s < 2.05, *p'*s > 0.163). This result was again not influenced by significant differences in pre‐shock activity levels (ANOVA model: Genotype_2_ × Intensity_5_; main effect of genotype and interaction between genotype and trial: *F'*s < 1.8, *p'*s > 0.189).

However, given the responses seen across repeated footshock presentations in Experiment 2 (Figure 4A), we then explicitly compared the responses to the first and second 0.4 mA intensity footshocks presented as part of our ascending/descending footshock sensitivity protocol. Indeed, closer inspection of the data revealed that successive delivery of the stronger aversive footshocks led to a greater reactivity in the 5‐HTTKO mice compared to the wildtype mice on the second of these shock presentations. Specifically, 5‐HTTKO mice significantly increased their reactivity from the first to the second presentation of the 0.4 mA footshock (main effect of trial for KO Δactivity: *F*(1,29) = 6.6, *p* = 0.016), and a significantly stronger reactivity could then be seen in these mice compared to wildtype mice on this second presentation (main effect of genotype for the Δactivity to the second 0.4 mA footshock: *F*(1,29) = 6.2, *p* = 0.018; Genotype_2_ × Trial_2_ interaction: *F*(1,29) = 4.5, *p* = 0.042). Again, this result was not influenced by significant differences in pre‐shock activity levels (ANOVA model: Genotype_2_ × Trial_2_; main effect of genotype and interaction between genotype and trial: *F'*s < 2.1, *p'*s > 0.156). This difference in footshock reactivity resembles the observation with repeated presentations of 0.3 mA shocks during C1 of Experiment 2. Thus, although the genotypes do not differ in their unconditioned response to the very first footshock, subsequent footshock deliveries elicited stronger reactivity in 5‐HTTKO mice.

## 4 Discussion

Here we used 5‐HTTKO mice as an experimental model of long‐term increased 5‐HT function and assessed fear learning, a widely used model of emotional learning in rodents. Our results show that 5‐HTTKO mice can show both more and less fear than wild‐type controls depending on the particular situation.

### 4.1 5‐HTTKO Mice Exhibit Increased Levels of Unconditioned Anxiety

In agreement with several previous studies (e.g., [29, 31]), 5‐HTTKO mice exhibited increased levels of unconditioned anxiety (Experiment 1). This was evident on the elevated plus maze and in the hyponeophagia test, both of which capitalise on approach‐avoidance conflicts, with 5‐HTTKO mice exhibiting increased avoidance behaviour consistent with increased anxiety. This is also consistent with a wealth of studies that demonstrate increased unconditioned anxiety when 5‐HT levels are increased (e.g., [13, 15, 58, 59]) and, conversely, reduced levels of anxiety as a result of reduced 5‐HT [12, 30, 31, 33]. An increase in anxiety in the 5‐HTTKO mice could also explain the increased reactivity to repeated presentations of noxious shock stimuli, despite no initial difference in the unconditioned response to the very first shock presentation, or in the threshold for footshock reactivity (Figure 4). Indeed, whereas conditioned fear typically reduces pain responses [60, 61, 62], it is well established that anxiety can exacerbate the response to painful, noxious stimuli ([63, 64, 65]; but see [66]).

### 4.2 5‐HTTKO Mice Exhibit Increased Learning About Cues Which Predict the Absence of Threat

5‐HTTKO mice were faster to discriminate between a cue that reliably predicted footshock and a cue that predicted the absence of footshock (Experiment 2). Notably, this enhanced discrimination performance in the knockout mice did not reflect faster learning that the CS+ cue was associated with footshock. Compared to pre‐CS baseline levels, the rate of increase in freezing to the CS+ cue did not differ between genotypes. Instead, this enhanced discrimination performance in the present study was driven by faster learning that the CS− cue signals the absence of footshock and thus provides relief from the fear/anxiety generated by the Pavlovian cues of the experimental context [67]. This result is slightly different to our previous study which used a similar fear discrimination learning paradigm involving both CS+ and CS− cues but which also included a day of pre‐exposure to the auditory cues prior to any conditioning [38], and which found enhanced learning about both the CS+ and CS− cues in the 5‐HTTKO mice. However, the present study is consistent with earlier studies that reported no differences in Pavlovian fear conditioning in 5‐HTTKO mice using single cue protocols which featured only a CS+ stimulus (e.g., [32, 68]).

Likewise, the 5‐HTTKO mice were also faster to learn that the CS+ cue no longer predicted footshock during subsequent extinction sessions, and thus were faster to reduce their levels of fear to this threat cue (Figure 2). This result appears to be at odds with previous studies in 5‐HTTKO rodents which have reported either reduced extinction [69, 70] or reduced extinction recall of fear memories [32, 68], leading to higher (and not lower) levels of fear in 5‐HTTKO mice in these experiments. Notably, however, facilitated fear extinction in mice has also been seen following chronic SSRI treatment regimens [44], demonstrating that increasing 5‐HT levels can reduce fear levels during extinction, although again it is worth pointing out that other rodent studies report either no alteration or an impairment of extinction learning with SSRIs [71, 72]. There are a number of procedural differences between these different studies investigating extinction, all of which might contribute to the different observed outcomes (e.g., the strength and duration of the US, the presence of a CS− cue, the salience of CS+, CS− and contextual cues, and the specific fear conditioning training protocol adopted). These different outcomes across studies illustrate the potential complexity of the psychological processes that are in play during fear extinction studies which belie the procedural simplicity of the behavioural task. Nevertheless, the 5HTTKO mice in the present study exhibit reduced fear, both to the CS− cue during conditioning and to the CS+ cue during extinction.

### 4.3 Increased Inhibitory Learning Reduces Fear in 5‐HTTKO Mice

It is well established that rather than degrading the original association between the CS+ and footshock, extinction involves new learning that in the current state the CS+ cue no longer accurately predicts the aversive outcome [73, 74]. Associative learning theories have posited that during extinction, an inhibitory association is formed between the target CS+ cue and the aversive outcome, in addition to the pre‐existing excitatory association between the CS+ and the shock US ([73] but see also [75]). Thus, the excitatory association provides a ‘danger’ meaning, whereas the inhibitory association provides a ‘safety’ meaning [55, 76, 77, 78]. The inhibitory association is activated by the extinction context, which plays the role of a negative feature or occasion setter and thereby suppresses activation of the shock US representation, thus reducing fear.

Notably, a similar mechanistic account could also explain the safety or relief provided by the CS− cue during discrimination learning. In this case, the context is an ambiguous predictor of footshock, with both excitatory and inhibitory associations to the aversive outcome. Now it is the CS− cue that acts as the occasion setter or safety signal which activates the “context‐no US” link and thus reduces fear. Alternatively, it is possible that the CS− could acquire a direct inhibitory association with the shock US. This could be investigated using either a summation test or a retardation of acquisition test [79], although this is beyond the scope of the present study. Nevertheless, both fear extinction and the development of safety/relief with a Pavlovian CS− cue require inhibitory associations to form between cues and potential aversive outcomes.

### 4.4 A Role for Plasticity

So why might increased levels of 5‐HT in 5‐HTTKO mice lead to enhanced fear extinction and/or safety learning in the present studies? And how can we relate this to the well‐established idea that increasing 5‐HT facilitates emotionally relevant learning by increasing plasticity processes in brain regions like the hippocampus and amygdala [4, 44]? An alternative way to conceptualise the formation of inhibitory associations, which borrows heavily on associative learning theory [80, 81, 82, 83], is to consider priming mechanisms whereby the memories of associations between stimuli are temporarily activated or tagged as relevant to the current situation. In turn, this priming of relevant memories could lead to better inhibitory learning about cues which predict that the threat won't occur, such as during safety learning or during extinction (i.e., enhanced inhibitory learning; [84, 85]). We have argued elsewhere that synaptic plasticity in the hippocampus may support the priming of memories [35, 86]. This could include both self‐generated priming (based on recent experience) and retrieval‐generated priming (based on associative recall) of both the sensory and emotional components of memories [81, 82, 83]. Furthermore, deficits in hippocampal synaptic plasticity can result in selective deficits in inhibitory learning [87]. Thus, if 5‐HT increases plasticity processes in the hippocampus (and particularly in the ventral hippocampus), then this could increase the priming of emotionally‐relevant memories [88, 89, 90]. This could then facilitate inhibitory learning for cues that predict the absence of threat. In this way, 5‐HT could promote emotionally relevant inhibitory learning that the threat likelihood is reduced. This could provide a potential mechanism for the improvement in mood seen with SSRIs in some patients, but only if the environmental context was favourable [5, 91]. In principle, these priming mechanisms could also facilitate the processing of positive occasion setters which could act to increase fear, for example during the CS+ cue in our previous study [38], again illustrating the importance of environmental cues in determining the emotional consequences of increased 5‐HT.

### 4.5 5‐HTTKO Mice Exhibit Increased Generalisation of Fear Across Contexts

Moreover, 5‐HTTKO mice exhibited increased generalisation of fear memory for ambiguous aversive cues (predicting footshock on 20% of trials) when tested in a novel context, which was different from the conditioning context (Experiment 3). In this experiment, mice were explicitly trained with an ambiguous CSa cue that was associated with both footshock and the absence of footshock. Thus, both excitatory and inhibitory associations will likely have formed between the CSa and shock representation. In this scenario, the contextual cues, which can act as occasion setters to either activate or suppress the inhibitory association between target cue and shock representation, become important for determining the expression of learning and behaviour.

For example, for wildtype mice, the conditioning context acts as a positive feature or occasion setter, which can release the excitatory CSa‐shock association, leading to the expression of fear in response to CSa presentations, relative to both pre‐CS levels and the CS− cue. In contrast, when wildtype mice were transferred to a novel, neutral context during the FMR session they now exhibited no fear to the CSa cue relative to the CS− cue. In this novel context, and thus in the absence of the conditioning context cues (i.e., in the absence of the positive occasion setter to suppress the inhibitory link between CSa and footshock), there will now be no freezing to this stimulus during recall. Importantly, this is a very different scenario from Experiment 2 with a fully predictive cue during which only CS+/shock associations will have formed during training. This learning, involving only excitatory associations, transfers very efficiently to a novel, distinct context during fear memory recall in both KO and wildtype mice [73, 74]. Therefore, the lack of any inhibitory association will favour generalisation of fear expression to the fully predicting CS+ cue across contexts, whereas with ambiguous cues for which there are both tone‐shock and tone‐no shock memories, the contextual cues become important for determining the nature and extent of the memory that is retrieved.

So why might 5‐HTTKO mice exhibit increased generalisation of fear memory for an ambiguous cue in a novel context? One possibility is that the inhibitory association between the CSa and footshock forms less well during training, leaving only the excitatory CSa‐shock link. However, this seems unlikely given the apparent increased propensity for inhibitory learning in the 5‐HTTKO mice during both extinction and safety/relief learning in Experiment 2.

Alternatively, the higher levels of anxiety seen in the 5‐HTTKO mice (Experiment 1) could provide sufficient contextual or internal state‐dependent cues so as to increase priming of fear memory expression, even in an otherwise novel context. More specifically, the high levels of anxiety in 5‐HTTKO mice could act as the positive occasion setter and release fear expression to the CSa. Thus, internal state cues (i.e., high levels of anxiety) might act as very important occasion setting cues for controlling the expression of fear memories across contexts. In 5‐HTTKO mice, a persistent state of high anxiety might result in their being less of a perceived difference between training and testing contexts, and thus the expression of the CSa fear memory will generalise more effectively across contexts as was seen in Experiment 3.

Indeed, there are important precedents whereby internal state can play a key role in regulating memory retrieval [92, 93], including for inhibitory associations and the expression of emotional memories. For example, when fear extinction is conducted in the presence of anxiolytic drugs, such as diazepam (Valium), chlordiazepoxide (Librium) and alcohol, renewal of fear then occurs when the animal is subsequently tested in the absence of the drug [94, 95]. Thus, drug‐induced changes in anxiety levels can act as internal state cues to influence the retrieval of inhibitory associative links and modulate fear memory expression. In a similar way, high levels of anxiety in 5‐HTTKO mice could provide the internal state cue to act as a positive occasion setter and facilitate the priming of fear memory for the CSa cue, even in a novel context. In this way, higher levels of anxiety due to increased levels of 5‐HT could increase the generalisation of fear memory expression across contexts.

### 4.6 Conclusion

Numerous accounts of the effects of long‐term increases in 5‐HT function posit an increase in neural plasticity which facilitates learning and responsivity to environmental cues ([1, 3, 4, 5, 6, 7, 42, 96]). It has been suggested that this learning represents “a double‐edged sword,” leading to either better or worse mood depending on the background environment [3]. Our results support the idea that loss of 5‐HTT function, and corresponding long‐lasting increases in synaptic 5‐HT availability, can indeed enhance emotionally relevant learning. This might include an increased priming of threat‐relevant memories, which could enhance inhibitory learning about threat‐relevant cues (e.g., safety signals and fear extinction learning). This, in turn, might be relevant to the improvement of mood in clinical situations following treatment with antidepressant drugs, including SSRIs [44, 67, 97], but only provided the environment is favourable [3, 42]. Indeed, subchronic SSRI administration can facilitate extinction of discriminate aversive learning in healthy participants [98]. The requirement for patients to experience a favourable environment (and to integrate this new learning into their model of the world) might contribute to the delayed onset of any beneficial effects on mood in patients taking anti‐depressant medication [99]. These results are potentially consistent with a role for 5‐HT in promoting plasticity in brain areas like the hippocampus which might be important for priming of memories and their regulation by occasion‐setting cues. At the same time, however, increases in 5‐HT in the 5‐HTTKO mice were also associated with increased unconditioned anxiety and increased generalisation of ambiguous fear memories across contexts. This is potentially consistent with the increase in anxiety that is often reported in patients when first taking SSRI medication (e.g., [100]), and is also consistent with the increased generalisation that is commonly observed in patients with anxiety disorders [101, 102, 103]. Thus, depending on the background environment and the experiences of the individual, long‐term increases in 5‐HT may indeed prove to be a ‘double‐edged sword,’ leading to either more or less aversive memories.

## Ethics Statement

Experiments were conducted following the United Kingdom Animals (Scientific Procedures) Act 1986 Amendment Regulations (SI 2012/3039), under project licenses PPL 30/2561, and 30/3068, and approved by local ethical review for the University of Oxford.

## Conflicts of Interest

The authors declare no conflicts of interest.

## Supporting information


Figure S1.

Figure S2.

## Data Availability

The data that support the findings of this study are available from the corresponding authors upon reasonable request.
