# Supplementary material for: More and Less Fear in Serotonin Transporter Knockout Mice
Source: Genes Brain Behav. 2025 Feb 7;24(1):e70016. doi: 10.1111/gbb.70016 (PMC11803413; doi:10.1111/gbb.70016)
Supplement: Supplementary file 1 — Figure S1. [file GBB-24-e70016-s002.pdf]

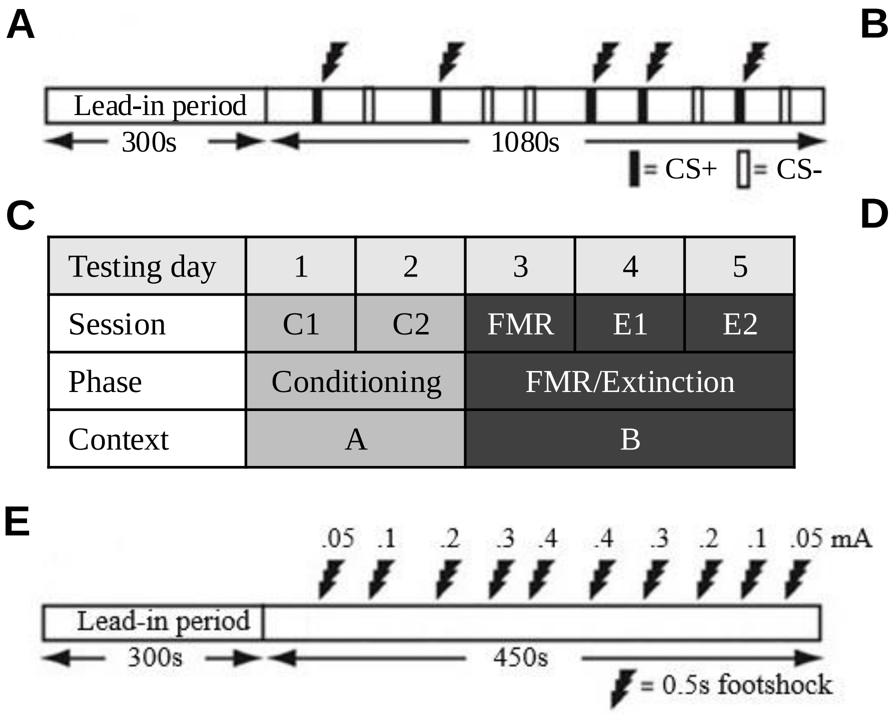

Supplementary Figure 1. Experimental procedures used in Experiments 2 and 3, and in the follow-up studies investigating genotypic differences in fear generalisation across different contexts and footshock reactivity. **(A)** and **(B),** diagrams of a conditioning session used in Experiments 2 and 3, respectively (Adapted from McHugh, S. B., Barkus, C., Huber, A., Capitão, L., Lima, J., Lowry, J. P., & Bannerman, D. M. (2014). Aversive prediction error signals in the amygdala. *Journal of Neuroscience*, *34*(27), 9024-9033). The black lightning marks indicate when an excitatory cue (CS+ or CSa) was followed by a footshock. Inter-cue intervals varied between 60 and 100s. (**C)** 5-day procedure used in Experiments 2 and 3, consisting of conditioning phase with two days (Conditioning days 1 and 2, or C1 and C2), followed by a FMR/Extinction phase over the next three days (day 3: Fear Memory Recall, or FMR; days 4 and 5: Extinction days 1 and 2, or E1 and E2). Technically, FMR and E1 and E2 were all extinction sessions and were administered in a context different from the one used during the conditioning phase (e.g. context B, if the subject was conditioned in context A). (**D)** 3-day procedure used in the follow-up study investigating genotypic differences in fear generalisation across different contexts. The procedure used was virtually identical to the one used in Experiment 3, with the difference that FMR was now administered in the same context where conditioning occurred. **(E)** Diagram of the experimental procedure used for the footshock reactivity test, where 10 footshocks were administered (adapted from McHugh et al., 2014). The order of presentation of the different footshock intensities used (0.05, 0.1, 0.2, 0.3 and 0.4mA) followed the one indicated in the diagram, where each lightning mark has the respective intensity used indicated next to it. Inter-shock intervals varied between 30 and 70s. Every footshock used in Experiments 2 and 3 and the follow-up studies lasted 0.5s.
