# Supplementary material for: More and Less Fear in Serotonin Transporter Knockout Mice
Source: Genes Brain Behav. 2025 Feb 7;24(1):e70016. doi: 10.1111/gbb.70016 (PMC11803413; doi:10.1111/gbb.70016)
Supplement: Supplementary file 2 — Figure S2. [file GBB-24-e70016-s001.pdf]

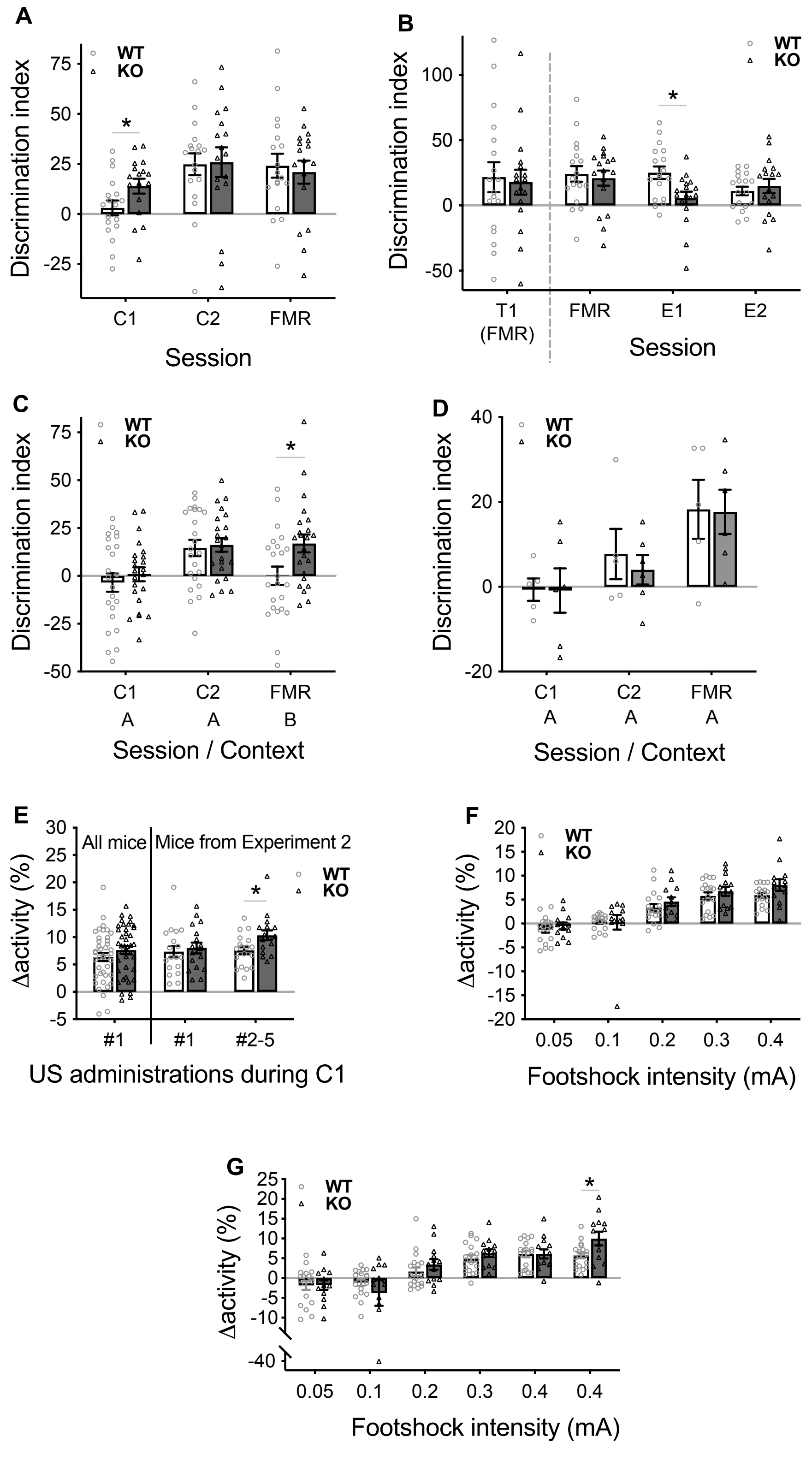

Supplementary Figure 2. A-D, Mean discrimination index results of Experiments 2 and 3, along with the follow-up study of Experiment 3, re-presented from Figures 1-3 and complemented with individual data points. **E-G**, Responses to the unconditioned stimulus in mice fear conditioned in Experiments 2 and 3 (E) and responses to five different shock intensities in mice subjected to a footshock reactivity test (F-G), re-presented from Figure 4 and complemented with individual data points. Mean ± SEM, *$p < .05$.
